# Update on infection prevention in the ICU

**DOI:** 10.1097/MCC.0000000000001313

**Published:** 2025-08-07

**Authors:** Filippo Medioli, Erica Franceschini, Cristina Mussini, Marianna Meschiari

**Affiliations:** a Department of Biomedical Sciences, Humanitas University, Pieve Emanuele; b Infectious Diseases Unit, IRCCS Humanitas Research Hospital, Rozzano, Milan; c Infectious Diseases Unit, Azienda Ospedaliera-Universitaria of Modena, University of Modena and Reggio Emilia, Modena, Italy

**Keywords:** antimicrobial resistance, healthcare-associated infections, infection prevention, intensive care unit, multidrug-resistant organisms

## Abstract

**Purpose of review:**

Healthcare-associated infections (HAIs) remain a critical challenge in intensive care units (ICUs) due to the high prevalence of invasive procedures, vulnerable patient populations, and the increasing threat of antimicrobial-resistant organisms (MDROs). This review synthesizes current evidence on infection prevention and control (IPC) strategies in the ICU setting, highlighting recent findings and innovations in this evolving field, particularly in light of the impact of the COVID-19 pandemic.

**Recent findings:**

The review outlines ten key IPC strategies for ICUs, categorizing them into horizontal (universal) and vertical (pathogen-specific) approaches. Recent literature emphasizes the importance of enhanced hand hygiene compliance through motivational interventions and feedback. The role of selective decontamination strategies remains debated, with evidence suggesting potential benefits in specific patient subgroups. Vertical strategies, including active screening for MDROs and per-pathogen bundles, are increasingly being tailored based on local epidemiology and pathogen characteristics. Studies suggest that de-escalating routine contact precautions for certain MDROs like Methicillin-resistant *Staphylococcus aureus* and Vancomycin-resistant *Enterococcus* may be safe in settings with robust horizontal measures. Conversely, intensified “search and destroy” strategies show promise in controlling carbapenem-resistant *Acinetobacter baumannii* outbreaks.

**Summary:**

Effective IPC in the ICU requires a multifaceted and adaptable approach, integrating both universal precautions and targeted interventions against specific pathogens. While consistent implementation of horizontal strategies like hand hygiene is foundational, tailoring vertical strategies based on local MDRO epidemiology and patient risk profiles is crucial. Future research should focus on harmonizing IPC policies, optimizing screening methods, and evaluating the long-term impact of combined IPC and antimicrobial stewardship programs to improve patient outcomes and mitigate the spread of antimicrobial resistance in critical care settings.

## INTRODUCTION

Healthcare-associated infections (HAIs) continue to pose a significant threat, particularly among patients admitted to intensive care units (ICUs). This elevated risk is primarily attributed to the high prevalence of invasive procedures, immunosuppression, multiple comorbidities, frailty, and advanced age in this population [1]. Critically ill patients are characterized by both intrinsic and extrinsic risk factors, which significantly narrow the window of opportunity for effective infection prevention. Consequently, there is a pressing need to intensify and tailor infection prevention strategies to the unique vulnerabilities of ICU patients [2]. Despite progress in reducing device-related infections, ICU-acquired infection rates remain high. In 2021, 10% of ICU patients developed pneumonia, 8% bloodstream infections (BSIs), and 4% urinary tract infections (UTIs), most linked to invasive devices: 60% of pneumonias to intubation, 38% of BSIs to catheters, and 97% of UTIs to urinary catheters. The ongoing emergence of novel antimicrobial resistant organisms (MDROs) adds a layer of complexity to ICU infection management, undermining therapeutic effectiveness and contributing to poorer patient outcomes. In ICU settings, *Staphylococcus aureus* isolates were oxacillin-resistant (MRSA) among 15% of cases and 7% of *Enterococcus* spp. were glycopeptide-resistant. Resistance to third-generation cephalosporins was reported in 20% of *E. coli* isolates, 42% of *Klebsiella* spp. isolates and 46% of *Enterobacter* spp. isolates. Carbapenem resistance was reported in 12% of *Klebsiella* spp. isolates, 30% of *P. aeruginosa* isolates and 85% of *Acinetobacter baumannii* isolates. Our growing knowledge of the interactions between critically ill patients and their microbiota, coupled with more sensitive microbiological techniques, is driving a re-evaluation of how we define HAIs and the optimal approaches for diagnosis, treatment, and prevention in the ICU [1]. The COVID-19 pandemic further worsened this already concerning epidemiological landscape, mainly because of compromised infection prevention and control practices (IPC) [2]. Several factors could have contributed to this deterioration in the ICU setting: difficulties for healthcare-workers (HCW) in adhering to standard IPC precautions; shortages of certain medical equipment; focus of on self-protection (e.g. universal gloving practices) rather than on preventing cross-transmission between patients; overcrowded facilities and possible staff shortages leading to low HCW-to-patient ratios; inadequate IPC training and discontinuation of IPC target policy such as active surveillance of MDRO-positive patients.

ICUs represent the tip of the iceberg of the MDR crisis and serve as pivotal settings for IPC efforts. Due to the high concentration of vulnerable patients and complex care procedures, ICUs act as amplification points for antimicrobial resistance (AMR) and HAIs. As such, strengthening surveillance and implementing targeted IPC strategies within ICUs are critical steps toward mitigating the spread of AMR across the broader healthcare system.

IPC practices comprise a comprehensive set of interventions aimed at reducing HAIs and are guided by recommendations from major professional bodies, including the Association for Professionals in Infection Control and Epidemiology (APIC), the Healthcare Infection Control Practices Advisory Committee (HICPAC), and the European Society of Clinical Microbiology and Infectious Diseases (ESCMID) [5–7]. Notably, a recent modelling study estimated that enhancing IPC programs in low- and middle-income country (LMIC) healthcare settings could prevent at least 337 000 (95% CI: 250 200–465 200) AMR-related deaths annually [8].

However, the available evidence for implementing these practices in different settings, and especially in the intensive care setting, remains fragmented, with recommendations based on studies of nonuniversal applicability or external validated. This review provides an overview of the main IPC measures currently deployed in the ICU setting, as well as the innovations in this field. 

**Box 1:**
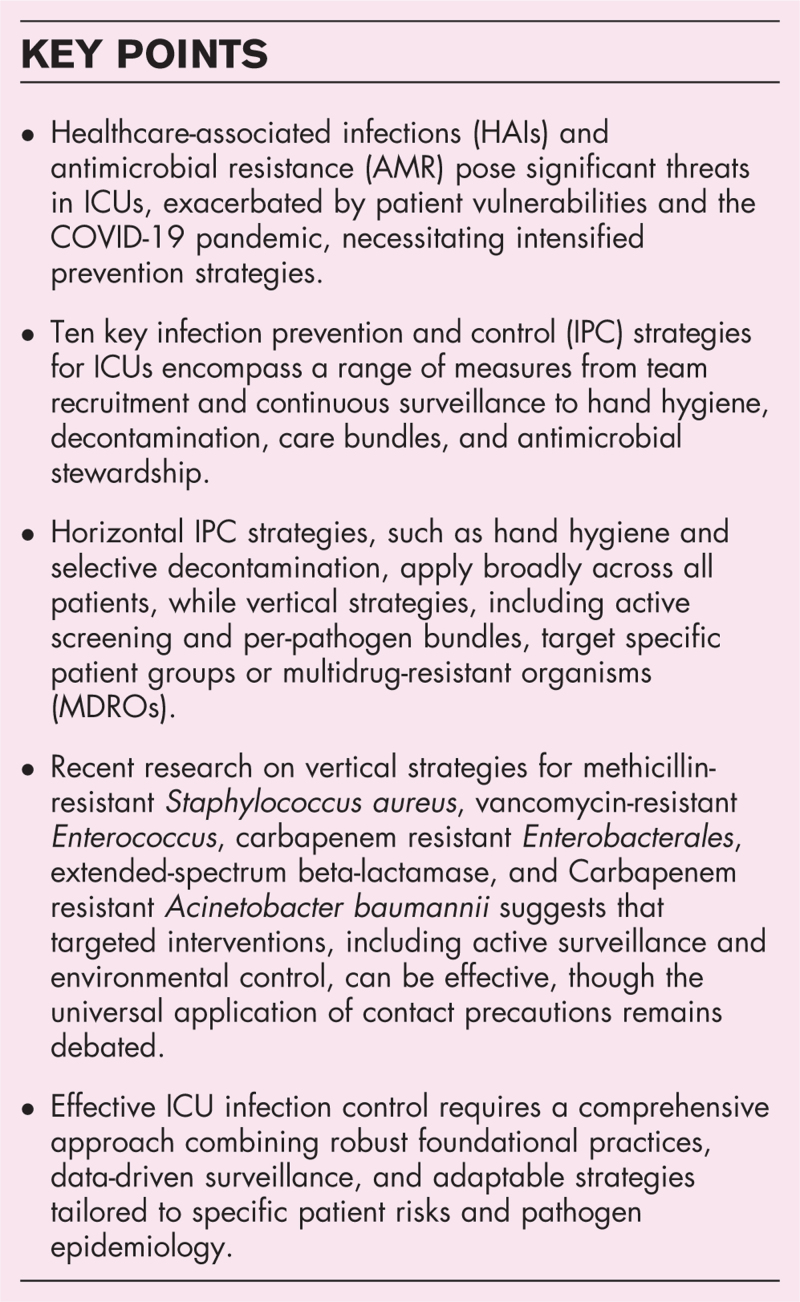
no caption available

## TEN KEY INFECTION PREVENTION AND CONTROL STRATEGIES FOR INTENSIVE CARE UNITS

Strategies for infection prevention and control in an ICU setting are multifaceted and involve several key components:

(1) Recruitment of the infection control team and their facilitators or stakeholders: Interdisciplinarity is crucial for the implementation of IPC programmes. A program leader with strong organizational and interpersonal skills must be appointed to manage the multidisciplinary work group and apply behavioural change. Additionally, in ICU, it is important to establish a network of internal champions/stakeholders who can monitor progress in real time and maintain high levels of attention through regular feedback [3].
(2) HAIs and alert organisms’ continuous HAIs surveillance: The minimal requirement for HAI-surveillance of ICU-acquired infections is to include bloodstream infection (BSI) and/or pneumonia (PN). In addition, a real-time automatic detection system for monitoring MDROs, along with regularly updated cumulative antibiograms incorporating all ICU microbiological isolates, is essential [4,5].
(3) Hand hygiene: enhanced performance feedback to improve hand hygiene compliance in ICU represents the cornerstone of all IPC strategies [6–8].
(4) Active screening strategies: In settings with a high prevalence of multidrug-resistant (MDR) pathogens, active surveillance cultures (ASCs) can play a crucial role in anticipating invasive infections and guiding targeted, vertical prevention strategies. ASCs have been performed universally or targeted to special group of patients. ASCs has also been used to guide appropriate antimicrobial therapy [9,10]. An individualized approach is presented in Table 1.
(5) Contact isolations: The benefit of universal glove and gown especially in ICU setting is controversial. Individual hospitals should consider the intervention based on the local epidemiology, patient vulnerability, effect sizes, compliance to standard precaution, resource and cost of instituting the intervention [11]. See Table 1 for details.
(6) Decontamination strategies: universal daily bathing with 2% chlorhexidine-impregnated washcloths for ICU patients to reduce the incidence of healthcare-associated infections (HAIs) due to both gram positive and gram negative MDROs. Conversely, target decolonization strategies direct on patients’ care should be tailored to patients’ individual risk profiles, including comorbidities (immunocompromised or transplant), clinical severity (ECMO, bowel hypoperfusion) and microbiota composition. A personalized approach, discussed in the paragraph below, should be strongly recommended.
(7) Environmental disinfection: enhance cleaning and disinfection with standard products and external monitoring should be preferred to no-touch decontamination (NTD) technologies. Conversely, NTD could be usefully used in terminal or discharge cleaning to reduce staff workload and limit their contact with reservoirs of infection [12^▪▪^].
(8) Care bundles: Infection control bundles in the ICU are structured multiple sets of evidence-based practices designed to prevent HAIs. These bundles may target specific types of ICU-related HAIs, such as ventilator-associated pneumonia, catheter-associated urinary tract infections, and central line-associated bloodstream infections, or be tailored to high-priority MDROs. The effectiveness of each bundle hinges on proper implementation and ongoing feedback to frontline staff [13^▪▪^].
(9) Education and training: Especially in ICU setting use of health-care simulation (HCS) for medical education has become more prevalent. Because patients in the ICU require invasive procedures and efficient interprofessional team performance in a high-stakes environment fraught with potential complications, HCS is particularly well suited to safely learn and practice both technical and team skills needed for critical care practice [14].
(10) Antimicrobial stewardship (AS): IPC and AS requires interdependent and coordinated action across multiple and overlapping disciplines and clinical settings. Deliberate strategic relationship-building actions will be required of IPC and AS program leaders to bring groups together to achieve the larger purpose of keeping patients safe from infection and ensuring that effective antibiotic therapy is available for future generations [15^▪^].

**Table 1:** Priority of personalized IPC (active screening and contact precaution strategies) based on patient, pathogen and infrastructure characteristics

	Low priority	Medium priority	High priority
Patient characteristics	• Healthy • Asymptomatic carriers • Low extrinsic health-care related risk • Short ICU (< weeks)	• Extensive medical interventions • Multisite colonization • Previous hospitalisation • Prolonged stay in the ICU	• Symptomatic infection • Immunocompromised patient • High intrinsic healthcare-related risk • Underlying comorbidity conditions
Pathogen characteristics	• Low mortality rate • Low transmission rate • Lower environmental stability (<1 day) • Endemic • Multidrug resistant (MDR)	• Self-limiting outbreaks • Moderate mortality rate • Moderate transmission rate • Moderate environmental stability (>1 week) • Extensively drug-resistant (XDR)	• High transmission rate • High environmental stability • High mortality rate • Epidemic • Pandrug-resistant (PDR)
Hospital characteristics	• High hand-hygiene compliance (>70%) • Audited IPC programme • Single room available	• Medium hand-hygiene compliance (>50%) • Limited IPC programme • Limited single rooms	• Low hand-hygiene compliance (<50%) • Open-space intensive care unit • Overcrowding

In general, IPC strategies could be classified in horizontal and vertical strategies. Horizontal refer to broad, system-wide measures that aim to prevent infections across all patients in the ICU. These strategies are typically based on best practices and evidence-based guidelines that apply universally to all patients, regardless of their specific risk factors. Horizontal strategies aim to reduce the general burden of infection through standard infection prevention measures. Vertical Strategies focus on tailored interventions for specific groups of patients, pathogen or medical procedure. These measures tend to be customized depending on the patient's risk profile or colonization status and should be adapted on different transmission pathway for each MDROs. Both horizontal and vertical IPC strategies are essential in the ICU setting. Horizontal strategies form the foundation of infection prevention, creating a broad and standardized approach to care. Vertical strategies, on the other hand, focus on tailoring interventions to the unique needs of critically ill patients, addressing specific risks and vulnerabilities. Combining both approaches ensures a comprehensive and effective strategy to minimize healthcare-associated infections in ICUs [16^▪▪^].

The second part of this review will focus on the most recent and debated issues in infection prevention and control (IPC) within the ICU setting.

## HORIZONTAL STRATEGIES

### Hand hygiene and glove usage

Hand hygiene stands out as the most effective factor in controlling nosocomial infections, leading to reduced costs, complications, and mortality rates. Nonetheless, the hand hygiene compliance rate (CR) remains low [6]. COVID-19 pandemic furtherly jeopardized those standard practices especially within Critical Care setting and ICUs, leading to a further reduction in compliance [7,8].

In this context, educational and motivational interventions appear to be the most effective for restoring functional compliance and reducing the transmission of multidrug-resistant organisms (MDROs): Abbasi *et al.* in 2024 assessed the effectiveness of motivational interventions on the rate of hand hygiene compliance in ICU nurses. Three study groups were formed: one received the motivational interview, the second a teach-back intervention, and the third was the control group, that received only the usual hospital teaching in hand hygiene. In group 1, hand rub use rose from 8% to 18.5%, and hand washing from 1.5% to 22%. Group 2 saw increases from 4% to 19.5% in hand rub and 3.5% to 17% in hand washing. Conversely, the control group showed no statistically significant change in hand hygiene compliance [17].

Namely, as the World Health Organization (WHO) in 2009 defined five moments for hygienic hand disinfection (HD) in their “My five moments for hand hygiene” (M5M) campaign [18], Siebers *et al.* in 2023 investigated the incidence of HD opportunities and the CR during the treatment of critically ill patients. Their results underline that, even though 100% compliance with hand hygiene (HD) may be unrealistic, current rates remain too low. Improvements should prioritize aseptic procedures that combine the lowest compliance with the highest procedural risk for the patient. Considering that Healthcare Personnel (HCP) often use gloves when hand hygiene is indicated, incorporating glove disinfection strategies into daily practice could enhance patient care [19].

Insufficient time, high workload, and understaffing tighter with gloves use are important barriers to hand hygiene adherence. The need for hand hygiene before donning gloves or direct alcohol-based glove disinfection remains an unresolved issue particularly in critical-ill settings requiring several sequential “opportunities” for hand hygiene [20]. Applying alcohol-based hand rub (ABHR) directly to gloved hands. Kerri *et al.* recently published a randomized control trial demonstrated that compared with usual care, contamination of gloved hands was significantly reduced by applying ABHR directly to gloved hands but statistically higher than the gold standard. Another important study by Lee *et al.* showed the reuse of gloves increased residual microbial colonies and potential for transmission of multidrug-resistant organisms, even after using alcohol-based hand rub. These findings seem to underline that direct ABHR glove disinfection in ICU should be limited to specific situations such as high workload, emergency conditions and limited to single patients care. Otherwise, gloves should be changed regularly to prevent perforation and maintain effectiveness in infection control.

### Selective oral and digestive decontamination

Selective decontamination of the oropharynx (SOD) and the digestive tract without or with systemic antibiotics (SDD) remains controversial.

Oral care is ICU essential to prevent endogenous bacteria colonization and subsequent infections. The Infectious Diseases Society of America (IDSA) and more recent recommendation published by Rosenthal *et al.* suggest providing regular oral care, including toothbrushing or using gauze if the patient has no teeth, as an essential practice to lower VAP rates [21,22^▪▪^]. Controversial exist on chlorhexidine (CHG) oral care. Dale *et al.* observed no benefit for de-adoption of CHG and implementation of an oral care bundle on ICU mortality, oral procedural pain, or time to extubation [23]. Later, a meta-analysis including 10 RCTs, concluded that while CHG effectively reduced VAP, the evidence on its impact on mortality was inconclusive [24]. Only one study compared the effects of selective digestive decontamination (SDD), selective oropharyngeal decontamination (SOD), and topical CHG on mortality in ICU patients through a network meta-analysis of RCTs. Both SDD and SOD were superior to CHG in reducing mortality, though the distinction between SDD and SOD was less clear [25^▪^].

Lately many studies explored the impact of SOD and SDD strategies in critically ill patients, also during the COVID-19 pandemic, showing promising results [26,27,29^▪^,30^▪^]. Martínez-Pérez *et al.* showed SDD reduced antibiotic use and resistant organism colonization in adults, but Kean *et al.* observed significant gut microbiome shifts in children with SDD, without impacting AMR genes or the oral microbiome. Young *et al.*'s analysis suggests SDD's benefits might be limited only to patients with acute brain injuries [29^▪^–31^▪^]. Crucially, Hurley highlighted discrepancies in mortality outcomes between SDD randomized controlled trials and cluster-randomized trials, raising ethical concerns about spillover effects. Brown *et al.*'s feasibility study addressed challenges in conducting large-scale SDD trials in children, further emphasizing the complexities of implementation [28,32].

Collectively, these studies indicate that while SDD holds promise in reducing antibiotic consumption and colonization, its impact on mortality and other outcomes may be context dependent. Significant ethical and methodological considerations necessitate careful design of future research, ideally using cluster-randomized trials, to definitively establish SDD's role and safety in diverse critically ill populations. Investigating how SDD alters the microbial composition of the digestive tract is crucial to optimizing its use and minimizing potential adverse effects, such as bacterial imbalances or the development of antibiotic resistance. Understanding these dynamics could lead to more personalized SDD approaches, tailored to patients’ individual risk profiles, including comorbidities and microbiota composition.

## VERTICAL STRATEGIES

### Active screening strategies

Early identification of MDROs carriers at hospital admission enables prompt infection control and guides initial antibiotic choices, specifically in ICU setting. Indeed, colonization is identified as the most significant risk factor for subsequent infection with the same MDRO, emphasizing the importance of detection, especially in immunocompromised patients [33,34,35^▪^,^36^] Among gram negative bacteria the risk of progression from carrier to infection differs significantly by bacterial species, setting and carbapenemase type [37^▪^].

Nevertheless, the quality of exiting evidence in favour of active surveillance screening, especially for MDR gram negative pathogens, is poor and solid conclusions cannot be drawn [38]. Systematic screening appears to increase broad-spectrum antibiotic use without improving patient outcomes, suggesting limited clinical benefit. Prediction models for MDRO colonization or infection show promise but currently lack sufficient validation and are prone to bias, limiting their clinical utility. Furthermore, the optimal site and method for MDRO screening, especially in vulnerable populations, require further investigation to balance detection rates with feasibility and minimize bias. As perianal screening is highly effective for detecting certain MDROs, combining room environment surveillance with nonperianal sites (nares, groin, hands) could improve detection of VRE and CRE carriers and mitigate this bias [39^▪▪^,^40^].

Both standardized national and international guidelines, improved risk stratification strategies, and further research into cost-effective and practical screening methods to effectively combat the spread of MDROs are needed, especially within dynamic context such as ICUs.

In conclusion, selective screening of high-risk patients or settings was generally more cost-effective, although universal screening was cost-effective in some cases where prevalence and transmission were high [41^▪▪^]. Finally, the results of a nation-wide Swiss survey of screening practice highlight the need for uniform national MDRO screening standards based on several factors including patient, hospital and pathogen characteristics [42]. Table 1 outlines the factors guiding prioritization strategies for IPC measures.

### Per-pathogen bundles

Health departments should strategically select a few key MDROs to guide their prevention plans and measures, ideally including multiple based on available resources and local epidemiology. Prioritizing MDROs in early epidemic stages is most effective for slowing spread, especially if prevalent in nearby areas. Other factors for selection include existing prevention programs, healthcare interest, and the feasibility of monitoring the plan's impact. This focus can be adjusted yearly based on evolving MDRO epidemiology within and around the region [43].

Table 2 summarizes the strongest evidence in the literature on targeting IPC interventions for specific MDRO pathogens in ICUs, focusing on transmission pathways, screening recommendations, surveillance sites, progression to infection among carriers, carriage at 12 months, and contact precautions [9,44^▪▪^,^45^,^46^,^47^^▪▪^,^48^]. The organisms included are methicillin-resistant *Staphylococcus aureus* (MRSA), vancomycin-resistant *Enterococcus* (VRE), extended-spectrum beta-lactamase (ESBL)-producing bacteria, carbapenem-resistant or colistin-resistant *Enterobacterales*, carbapenem-resistant *Acinetobacter baumannii* (CRAB), and carbapenem-resistant *Pseudomonas aeruginosa* (CRPA). The data presented aims to guide infection prevention and control measures tailored to each organism's specific risks and characteristics.

**Table 2:** Per-pathogen bundle of interventions

Organism	Main transmission pathway	Screening recommendation	Surveillance site	Progression to infection among carriers	Carriage at 12 months	Contact precautions
MRSA	Clonal /patient-to-patient/patient-to-HCWs	Universal in ICU Target for high-risk patients (i.e. presurgery)	Active infection site Nares, throat, perirectal, wound	15–20%	30–43% (median 9 months)	Yes (very low evidence)
VRE	Clonal /environmental source/ endogenous selection (dysbiosis)	Usually not recommended (high-risk patients: haematological and transplant)	Active infection site Stool, rectal and perirectal	2–8%	40–62% (median 6–7 months)	No (very low evidence)
ESBL	Antibiotic pressure/clonal transmission (i.e. *K. pneumoniae*)	Solid Organ Transplant and Colorectal Surgery Patients (haematological patients in general, at high-risk for febrile neutropenia)	Active infection site Rectal	3–25%	26–46% (median 6–7 months)	No (very low evidence)
CRE	Clonal /patient-to-patient/patient-to-HCWs/ antibiotic pressure	Universal in endemic settings Target for epidemic setting low-risk units	Perirectal or rectal alone or in combination with oropharyngeal, endotracheal, inguinal or wound	3–30%	23–48% (median 6–7 months)	Yes (recommended)
CRAB	Clonal /environmental source	Target for high-risk Units Universal for outbreaks	*Multisite:* Skin, Buccal mucosa Nares, oropharynx, groin, perianal area, wounds and device insertion site	30%	Not explicitly detailed (median 17–18 months)	Yes (strongly recommended)
CRPA	Antibiotic pressure /environmental source (water)	Usually not recommended Target for outbreaks	Rectal and endotracheal	45%	Not explicitly detailed (median 18–24 months)	No (very low evidence)

CRAB, carbapenem resistant *Acinetobacter baumannii*; CRE, colistin/carbapenem resistant Enterobacterales; CRPA, carbapenem resistant *Pseudomonas aeruginosa*; ESBL, extended spectrum beta-lactamases; HCW, health-care worker; MRSA, methicillin resistant *Staphylococcus aureus*; VRE, vancomycin resistant Enterococci.

Below are examples of recent bundle-based interventions targeting specific MDROs.

#### Methicillin resistant *Staphylococcus aureus* and vancomycin resistant enterococci (VRE)

SHEA/IDSA/APIC/AHA guidelines recommend active surveillance testing (AST) in acute-care hospitals to identify asymptomatic MRSA carriers for targeted infection control, including contact precautions and decolonization. Universal ICU decolonization is preferred over AST with contact precautions, while hospital-wide AST has shown significant MRSA reductions. AST is also useful in outbreaks, and HCP screening is advised if linked to clusters [49].

A recent study by Evans *et al.* demonstrated that removal active surveillance and contact precaution in high-risk epidemic ICU setting was associated with higher rate of MRSA-HAIs. Moreover, the implementation of a MRSA nasal screening for critically ill patients resulted in significantly shorter duration of anti-MRSA treatment without negative effect on patient outcomes [50].

Conversely, a study by our group in haematological patients identified risk factors for VRE colonization – such as vancomycin use and dysbiosis – which were linked to higher rates of VRE bloodstream infections and *C. difficile* infections, but not increased 30- or 90-day mortality. These findings underscore the importance of enhanced antibiotic stewardship in this population, rather than the implementation of pathogen-specific IPC measures [46]. Multicentre studies shown that discontinuing routine contact precautions (CP) for VRE and MRSA did not lead to a significant increase in either specific or overall, HAI rates, supporting the effectiveness of horizontal prevention strategies – such as strict hand hygiene – in infection control. One study further indicated that discontinuation of CP was particularly safe in settings with low baseline HAI rates and high adherence to hand hygiene practices [47^▪▪^,^48^]. Additionally, modelling from an ICU trial demonstrated limited impact of CP on MRSA transmission and no significant effect on VRE, despite its higher transmissibility.

In conclusion, the evidence suggests that discontinuing routine contact precautions for MRSA/VRE in certain patient populations and settings, especially when robust horizontal infection prevention measures are in place, does not lead to increased HAI rates.

#### Carbapenem resistant *Enterobacterales* and extended spectrum beta-lactamases

The main limitation in establishing clear evidence for IPC strategies targeting CRE is that most studies incorporate active surveillance cultures within bundled interventions, making it difficult to isolate and evaluate the independent impact of screening strategies. A recent systematic review addressing the role of active surveillance showed a decline of CRE infection and colonization rates after the implementation of screening strategies [51]. However, the Dutch hospital survey reveals significant inconsistencies in the application of contact precautions for carbapenemase-producing Enterobacterales (CPE) and extended-spectrum beta-lactamase-producing *Enterobacteriaceae* (ESBL-E) in non-ICU settings, highlighting a lack of policy harmonization that hinders consistent implementation and collaborative efforts [52^▪^,^53^,^54^]. In 2022 a retrospective study in Singapore over 4.7 years analysed 779 CRE patients, finding that only direct ward contacts clonal transmission decreased over time. Indirect ward and hospital contact remained risk factors for clonal transmission, and plasmid-mediated transmission, accounting for half of CRE spread, persisted. The study concludes that undetected CRE reservoirs challenge current infection control, necessitating new strategies to address plasmid-mediated transmission [55]. A cluster-randomized crossover study across 16 Dutch hospitals compared contact precautions in single versus multiple-bed rooms for preventing ESBL-producing Enterobacteriaceae transmission. In the per-protocol analysis of 463 index patients and 7093 wardmates, transmission rates were 4% in single rooms and 7% in multiple rooms, with a risk difference of 3.4% (90% CI −0.3 to 7.1). The study concluded that contact precautions in multiple-bed rooms were noninferior to single-bed rooms for preventing ESBL transmission, suggesting a potential broadening of infection control strategies [56]. While synergistic approaches like combined IPC and antimicrobial stewardship show promise in controlling MDR-GNB, and multicomponent strategies appear most effective, the inconsistent application of fundamental IPC measures across healthcare settings due to differing interpretations necessitates policy harmonization to optimize prevention and control efforts against these critical pathogens. Further research, particularly into bacteria-specific prevention methods and the sustained impact of combined strategies, is warranted.

In conclusion, implementing active surveillance for asymptomatic CRE carriage – using rectal or faecal screening in high-risk patients and tailored to local epidemiology – is strongly recommended, even outside the ICU. In low-prevalence settings, a targeted approach focused on high-risk areas, guided by periodic point-prevalence surveys, may represent the most effective strategy, with the flexibility to intensify IPC measures in the event of an outbreak.

### Carbapenem resistant *Acinetobacter baumannii*

CRAB poses a significant threat in hospitals with high mortality rates. Our group analysed studies on IPC strategies against CRAB in the last decade. The included studies, mostly outbreak reports in ICUs with up to 50% mortality, showed varied implementation of IPC measures like disinfection, contact precautions, and screening. While outbreaks were controlled, the heterogeneous IPC approaches depended on the setting. The review concludes that intensified ‘search and destroy’ strategies targeting both the environment and patients are the most effective for long-term CRAB elimination, confirming our experience with a 2018 CRAB outbreak in a 12-bed open-space ICU. A bundle intervention focused on reinforced hand hygiene and extended screening (including environment), universal contact precautions, and a one-time cycling radical environmental cleaning reduced ICU-CRAB incidence from 30.4 to zero cases per 1000 patient-days [57,58]. Ben-David *et al.* demonstrated that a phased intervention, culminating in multisite screening, significantly reduced CRAB incidence in an endemic hospital, emphasizing the importance of routine active surveillance. Martinez-Resendez *et al.* found that daily chlorhexidine gluconate (CHG) bathing in ICU patients reduced CRAB on environmental surfaces, suggesting this practice can limit environmental contamination, a potential source of transmission. Finally, the quasi-experimental study showed that rapid CRAB diagnosis using clinical specimen-direct loop-mediated isothermal amplification (LAMP) and subsequent early intervention significantly decreased CRAB infection incidence and transmission compared to standard culture methods [48,59,60]. Collectively, these findings underscore the need for comprehensive CRAB control strategies. The evidence suggests that a combination of proactive multisite screening, environmental control, and rapid diagnostics holds the key to effectively managing and reducing the burden of CRAB in ICU settings.

### Pseudomonas aeruginosa

ICU patients, especially those on mechanical ventilation, face a high risk of pneumonia, with *Pseudomonas aeruginosa* (PA) being a frequent cause. Patients can be colonized with PA on admission or acquire it in the ICU, with the gut and respiratory tract being potential reservoirs. The link between PA colonization at different body sites and subsequent pneumonia development is unclear due to limitations in prior studies. Recanatini *et al.* aimed to determine the incidence of PA ICU pneumonia (PAIP) and its independent association with early and ICU-acquired PA colonization in both the lower respiratory tract and perianal area. Their prospective study across 30 European ICUs enrolled 1971 mechanically ventilated adults, assessing PA colonization in the perianal and respiratory tracts at admission and twice weekly. PA-associated invasive pulmonary infection (PAIP) developed in 1.8% of patients, with colonization at admission or acquisition during ICU stay being common. Both perianal and respiratory PA colonization were independently associated with a four-fold increased risk of PAIP. The findings suggest that identifying and targeting PA colonization could be an opportunity for preventive interventions against PAIP in the ICU [47^▪▪^].

## CONCLUSION

This review underscores the multifaceted nature of infection prevention and control in the intensive care unit. The ten key strategies, encompassing both broad horizontal measures like hand hygiene and targeted vertical interventions for specific pathogens, highlight the complexity of minimizing healthcare-associated infections. Recent evidence emphasizes the importance of tailored approaches, such as selective decontamination and active screening, while also questioning the universal application of contact precautions. A combination of robust foundational practices, data-driven surveillance, and adaptable, pathogen-specific bundles, guided by local epidemiology and interdisciplinary collaboration, is essential to safeguard vulnerable ICU patients.

## Acknowledgements

*None. outside of the authors.*

### Financial support and sponsorship

*None.*

### Conflicts of interest

*There are no conflicts of interest.*

## REFERENCES AND RECOMMENDED READING

Papers of particular interest, published within the annual period of review, have been highlighted as:

▪ of special interest
▪▪ of outstanding interest
